# Arrays of Individual DNA Molecules on Nanopatterned Substrates

**DOI:** 10.1038/srep42075

**Published:** 2017-02-13

**Authors:** Roland Hager, Alma Halilovic, Jonathan R. Burns, Friedrich Schäffler, Stefan Howorka

**Affiliations:** 1 Center for Advanced Bioanalysis GmbH, 4020 Linz, Austria; 2 Institute for Semiconductor and Solid State Physics, Johannes Kepler University, 4040 Linz, Austria; 3 Department of Chemistry, Institute of Structural and Molecular Biology, University College London, London, England, United Kingdom

## Abstract

Arrays of individual molecules can combine the advantages of microarrays and single-molecule studies. They miniaturize assays to reduce sample and reagent consumption and increase throughput, and additionally uncover static and dynamic heterogeneity usually masked in molecular ensembles. However, realizing single-DNA arrays must tackle the challenge of capturing structurally highly dynamic strands onto defined substrate positions. Here, we create single-molecule arrays by electrostatically adhering single-stranded DNA of gene-like length onto positively charged carbon nanoislands. The nanosites are so small that only one molecule can bind per island. Undesired adsorption of DNA to the surrounding non-target areas is prevented via a surface-passivating film. Of further relevance, the DNA arrays are of tunable dimensions, and fabricated on optically transparent substrates that enable singe-molecule detection with fluorescence microscopy. The arrays are hence compatible with a wide range of bioanalytical, biophysical, and cell biological studies where individual DNA strands are either examined in isolation, or interact with other molecules or cells.

Single-molecule arrays are scientifically exciting and of considerable biotechnological interest^1^,^2^,^3^. They are composed of spatially ordered, individually discernable molecules such as DNA strands or proteins. Like conventional micro and nanoarrays, single-molecule arrays condense biomolecular assays into a parallel and highly miniaturized format to drastically increase the through-put of examination with the added benefit of reduced sample and reagent consumption. This is relevant in biosensing^2^ but also in the biophysical examination of molecules where their isolated, known position in arrays avoids interference from other nearby molecules^3^,^4^,^5^,^6^. Single-molecule studies also reveal biologically important heterogeneities in the molecular populations, and dynamic changes of molecules that are masked in conventional ensemble measurements^7^. Patterns of single molecules furthermore help study gene uptake by cells, and, cell activation when DNA aptamers achieve recognition of membrane proteins^8^,^9^,^10^,^11^,^12^,^13^,^14^,^15^.

Generating single-molecule arrays faces the experimental hurdle of handling individual DNA strands. For example, a AFM cantilever can locally deposit molecules onto gold surfaces^3^,^16^,^17^. An alternative is to adsorb from solution DNA strands onto structurally pre-defined, adhesive nanosites that are so small that only one molecule can bind. Chemically or electrostatically adhesive nanosites can be obtained via various top-down nanopatterning methods^3^,^18^,^19^,^20^,^21^,^22^,^23^,^24^,^25^ such as e-beam and UV lithography^26^,^27^,^28^,^29^,^30^,^31^,^32^, nanoimprint lithography^33^, and nanocontact printing^34^. Several of the patterns can be produced on optically transparent substrates which enablies read-out with widely used fluorescence microscopy, as shown for nanosites with multiple DNA molecules^31^. But binding individual strands in 1:1 stoichiometry has not yet been achieved due to the structurally dynamic nature of DNA strands and the resulting geometric mismatch to the defined and static nanosites. In this regard, flexible single-stranded DNA is more difficult than compacted DNA particles^2^,^35^,^36^ even though the former are of greater relevance in many biomolecular assays.

Here we fabricate arrays of individual, single-stranded DNA. Carbon nanoislands with highly tunable dimensions are written with an electron microscope. Using the size-exclusion principle, the DNA strands of biologically relevant gene-like length are bound to 50 nm-sized nanoislands. The glass substrates enable highly sensitive fluorescence detection to visualize single DNA strands at high throughput as well as high temporal and spatial resolution, thereby opening up many biomolecular assays. The nanopatterned substrates have a flat molecular and inert surface and are set to enable a wide range of cell biological studies. The generic fabrication route can vary the diameter of the nanoislands and is designed to be compatible with other DNA strands of different length.

## Results and Discussion

### Principle of forming single-molecule DNA arrays

**Figure 1:**
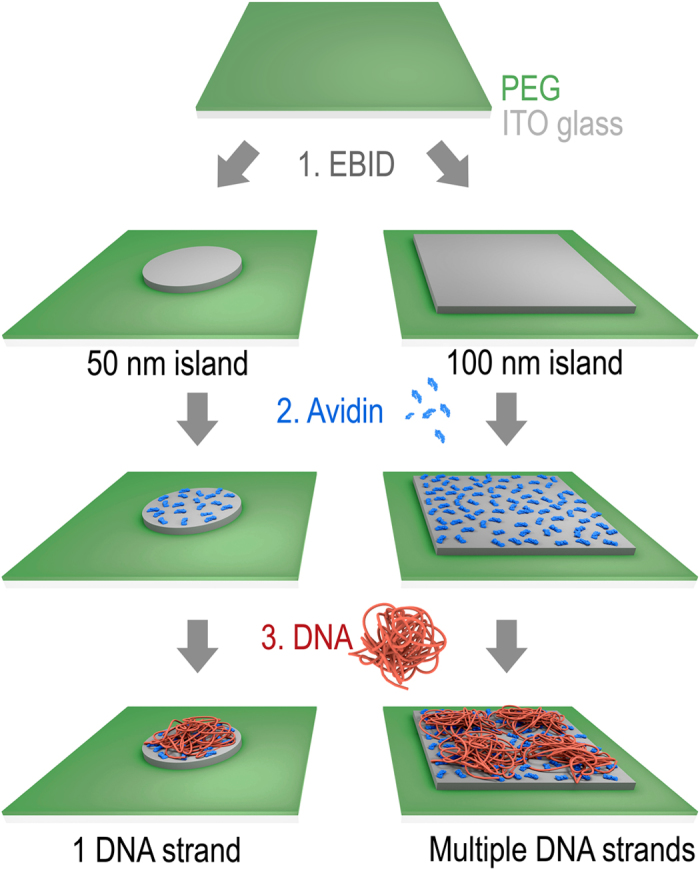
Scheme illustrating the generation of nanoarrays of individual DNA strands using 50 nm islands (left) and arrays with multiple DNA strands per 100 × 100 nm islands (right). The procedure involves (1) writing of carbon nanoislands (grey) of defined geometry via e-beam induced deposition (EBID), (2) coating of carbon islands with adhesive protein layer composed of avidin (blue), and (3) binding of individual DNA strands (red) to protein-coated islands. The binding is mediated by electrostatic attraction between negatively charged carbon nanoislands, positively charged protein, and negative DNA strands. EBID writing is performed on an ITO-glass substrate which is coated with a PEG film (green) to prevent non-specific binding of protein. For visual clarity, only one island each is shown, and the diameter of the bound, looped DNA strand was reduced to show the underlying carbon nanoisland. For visual simplicity, the number of DNA strands per 100 nm island is shown to be four which is lower than the experimentally determined number of approximately 10 DNA strands.

The fabrication of the single-molecule DNA array is schematically illustrated in Fig. 1. In step 1, islands composed of carbon (Fig. 1, dark grey) are written by deposition of carbon with an electron beam. Island shape, diameter and height are tunable via the electron dose as illustrated by disc-like nanoislands with 50 nm diameter and square islands with a side length of 100 nm. The nanoislands are written onto an optically transparent substrate which is covered with a surface passivating poly(ethylene glycol) (PEG) film (Fig. 1, green area). PEG prevents non-specific binding of biomolecules to non-target areas surrounding the islands in the two subsequent steps. In step 2, the negatively polarized carbon islands are selectively coated with a layer of positively charged avidin protein^31^,^37^. Avidin has a size of 4 × 5 × 6 nm. Consequently, many avidin molecules bind onto the larger nanoislands. In step 3, individual single stranded DNA molecules of a few kilo nucleotide length adsorb electrostatically to the nanoislands of 50 nm diameter. Single stranded DNA is a highly dynamic structure but can form very loosely structured nanoballs^38^ with hydrodynamic diameter in the range of 60 nm for the DNA selected in this study^39^. Given the matching dimensions between the DNA nanoball and the 50 nm nanoisland, only individual DNA strands are expected to bind to the discs. By contrast, islands of 100 nm are anticipated to adsorb multiple rather than individual DNA strands (Fig. 1). Binding of the looped and loose nanoballs to the flat substrate surface is thought to change their shape to a flattened hemisphere^38^. The influence of island height and completeness of the carbon films is also examined by varying the e-dose.

### Generation of arrays of carbon nanoislands

To generate nanoislands via e-beam writing (Fig. 1, step 1), we used glass slides coated with a 17 nm-thin layer of indium tin oxide (ITO). ITO dissipates electrical current during the e-beam writing, and is optically transparent to allow ultrasensitive fluorescence read-out of adsorbed biomolecules^40^. ITO was subjected to chemical surface modification to graft a PEG film^40^,^41^ which had the expected chemical identity and thickness, and a high lateral homogeneity and excellent passivation properties^42^.

The nanoislands were written via e-beam-induced deposition (EBID) of carbon (Fig. 1, step 1; see also Methods: Electron Beam-Induced Deposition)^43^,^44^. The process of EBID involves the localized destruction of the PEG film via electrical reduction and the deposition of the reduced carbon at the sites exposed to the e-beam^43^,^44^. EBID offers excellent control over the lateral position of the nanoislands, as well as their size and height, the latter by varying the electron dose. For our arrays, islands of two sizes were fabricated. (a) Disc-like nanoislands with a diameter of 50 nm to bind individual DNA strands (Fig. 1, left), and, (b) square islands with a side length of 100 nm to achieve in a control experiment binding of multiple DNA strands (Fig. 1, right). Furthermore, islands of different height were written to explore the role of island thickness in binding DNA strands; previous reports found that higher e-doses first lead to a compete carbon layer and then to a higher film thickness causing more binding of protein^27^,^31^,^45^.

The dimensions of the carbon nanoislands, as confirmed via atomic force microscopy (AFM), were in line with the nominal and expected size^31^,^37^. Examples of the AFM analysis of 100 × 100 nm are shown in Supporting Information, Figure S-1. The island dimensions were also studied as a function of e-dose. The dose, which ranged from 0.15 to 2.9 pC, did not alter the lateral size by more than 10% but more strongly influenced the height; a maximum dose led to an island thickness of 3 nm^31^,^37^. The pitch between the islands was set at 3.2 μm, i.e. above the diffraction limit to optically resolve the islands with fluorescence microscopy.

### Coating of carbon islands with an adhesive protein layer

**Figure 2:**
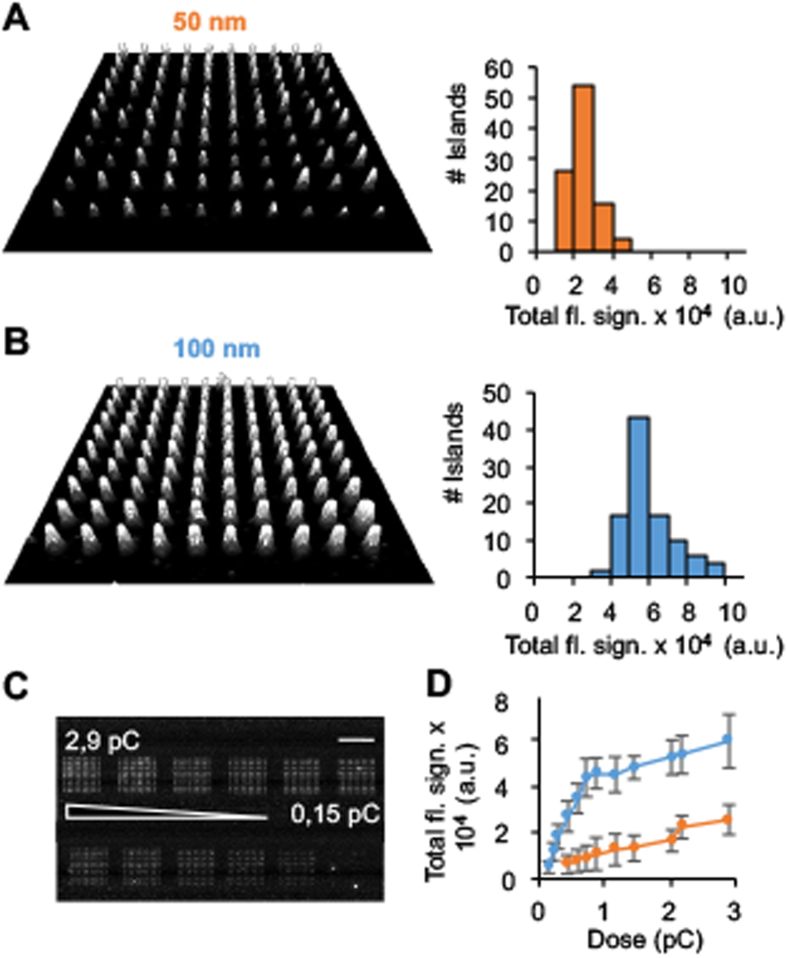
Avidin protein labeled with Cy3 binds to carbon nanoislands of (**A**) 50 nm diameter and (**B**) 100 nm × 100 nm side length. (**A**,**B**) Left panels: Fluorescence microscopic images of 10 × 10 arrays written with an electron dose of 2.9 pC. Right panels: Distribution of fluorescence of the Cy3-labeled proteins on islands. (**C**) Fluorescence microscopic image of a series of 10 × 10 arrays of 50 nm islands functionalized with avidin-Cy3. Islands within an array were written with the same electron dose while between arrays the dose was varied from 0.15 to 2.9 pC. Scale bar: 32 μm. (**D**) Total fluorescence signal per island vs electron dose of 50 nm and 100 nm islands. The averages and standard deviations were derived from four independent experiments comprising eight data sets.

Carbon nanoislands were biofunctionalized by incubating the array with avidin protein, followed by washing (Fig. 1, step 2). We used Cy3-labelled avidin (average number of 3.7 Cy3 dyes per protein) to demonstrate specific binding via fluorescence microscopy. We used a scanning method that combines ultrasensitivity and high spatial resolution with a high read-out speed to cover an area of 0.1 mm^2^ in a few seconds^46^. A typical fluorescence scan of a 10 × 10 array of 50 nm islands written at 2.9 pC is shown in Fig. 2A. All islands were covered with protein. The variation in brightness is in line with the stochastic binding of a small number of proteins; a maximum of approximately 90 closely packed protein molecules are calculated to fit onto a 50 nm island but the actual number of adsorbed molecules is lower. The distribution of Cy3-tagged avidin on the islands (Fig. 2A) had an average of 14900 ± 3500 total fluorescence counts (n = 400; n, number of spots) (N = 4; N, number of independent experiments). In line with expectations, control islands with 100 nm side length bound more protein (Fig. 2B, 51500 ± 8600 counts, n = 800, N = 4). If optical effects such as quenching of fluorophores are excluded, the fluorescence difference implies that 4.2-fold more protein adsorbed onto 100 nm islands that have a 5-times larger area (100 nm, 45600 ± 6600 counts; 50 nm, 10800 ± 700 counts; e-dose of 0.87 pC).

Varying the other dimension of the islands, that is their thickness, also tuned the apparent fluorescence intensity of the adsorbed protein, as shown for a series of arrays written at doses ranging from 0.15 to 2.9 pC (Fig. 2C). Following quantitative analysis (Fig. 2D), brightness increased steeply up to 0.9 pC for 100 nm islands followed by a shallower slope, which was also found for 50 nm islands. The steep dependence represents the formation of a contiguous film of carbon deposits within the 100 nm islands, and consequently binding of more protein. 50 nm islands do not show this stage as the same e-dose is concentrated on a 5-times smaller area leading to a contiguous film already at a low 0.2 pC. The other, shallow brightness increase found for both islands can be attributed to the further growth in island thickness, concomitant with addition of negative charges, and more protein binding^31^. The same bi-phasic dependence of fluorescence on e-dose was also found for islands of 300 nm side length (Supporting Information, Figure S-2). An additional explanation relies on the recently discovered distance-dependent quenching of fluorophores by ITO^47^ which is the substrate for our arrays. Following this explanation, fluorescence increases with island height as avidin-Cy3 is more distant from ITO. Avidin bound to the carbon islands retained its structure as suggested by its ability to recognize and capture the cognate ligand biotin (Supporting Information, Figure S-2). The fluorescence analysis also showed that binding of protein to the nanoislands vs the surrounding PEG-coated non-target areas was high and specific (Supplementary Information, Fig. S-3).

### Binding of few to individual DNA strands onto nanoislands

**Figure 3:**
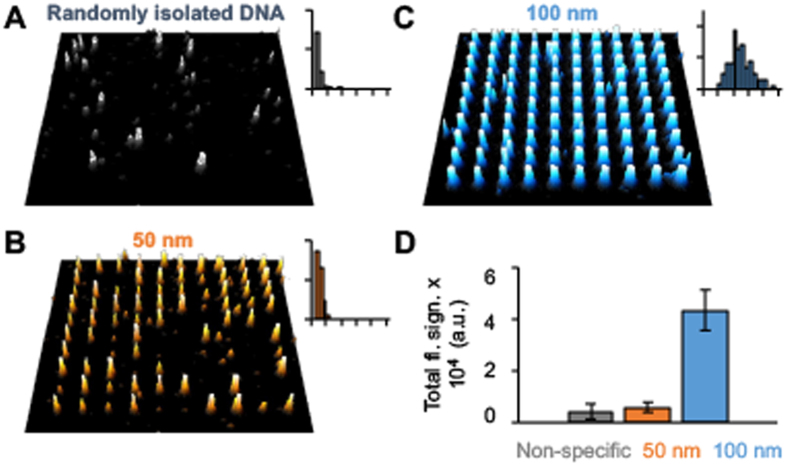
Cy3-labeled DNA strands bind electrostatically with 1:1 stoichiometry onto 50 nm avidin-coated carbon nanoislands while multiple DNA strands adsorb onto 100 nm islands. DNA was dissolved in TAE buffer supplemented with 14 mM MgCl_2_. (**A**) Fluorescence microscopic image of an unpatterned substrate with non-specifically bound labeled DNA strands. (**B**,**C**) Fluorescence microscopic images of arrays of 10 × 10 islands with (**B**) 50 nm diameter and (**C**) 100 nm side length written with an electron dose of 0.87 pC. Image size: 39 × 39 μm. The insets for A to C show the distribution of fluorescence on the islands obtained from the microscopy images. The x-axis scale is the same as in Figure 2. (**D**) Total fluorescence signal of individual non-specific bound DNA, and DNA-derivatized 50 nm and 100 nm islands.

The electrostatically adhesive 50 nm islands were exploited to bind individual DNA strands of negative charge (Fig. 1, step 3). We used single stranded DNA of 7249 nucleotides length which can form nanoballs with a hydrodynamic diameter of 50–60 nm in buffers with ionic strength of 0.05 to 0.1 M^39^,^48^. This hydrodynamic diameter of the nanoball matches the size of the smaller nanoislands and is expected to lead to a 1:1 stoichiometry when the DNA binds to the island. Upon binding to a substrate surface, the nanoball can adopt a flattened shape which is further compressed when imaged by AFM to yield an inverted cup with a height of 14.9 ± 4.3 nm and a diameter of 119 ± 22 nm (n = 20)^38^ (Supplementary Information, Fig. S-4). To facilitate fluorescence detection of the bound DNA strand, labeling with the Cy3 fluorophore was achieved by the covalent attachment at nitrogen 7 of the guanine base (Supplementary Information, Fig. S-5). DNA strands were labeled with multiple fluorophores to avoid issues that arise from bleaching of single fluorophores. The successful fluorescence tagging was confirmed by gel electrophoresis (Supplementary Information, Fig. S-5). The average number of Cy3 dyes per DNA strands was obtained by isolating individual molecules via non-specific binding onto non-arrayed substrates and subjecting them to fluorescence scanning (Fig. 3A). The average brightness of single strands was 4290 ± 2990 counts (n = 101) which corresponds to approximately 100 fluorophores per strand calculated from the known average brightness of individual Cy3 fluorophores, if other optical effects such as quenching is excluded. The fluorescence distribution of the Cy-3 labeled DNA (Fig. 3A) was in line with the stochastic number of labeled guanine bases within the strand.

To visualize the binding of individual Cy3-tagged DNA strands onto nanoislands, arrays were coated with avidin without fluorophore tag. Fluorescence microscopy (Fig. 3B) established that the majority of the 50 nm islands had bound Cy3-labeled DNA strands. The distribution of fluorescence signals on islands was narrow (Fig. 3B). Strikingly, the average fluorescence of 5920 ± 1960 cts (n = 388, N = 2) was almost the same as for the non-specifically bound DNA strands (Fig. 3D). This strongly supports the notion that individual DNA strands are bound to nanoislands. This interpretation is supported by additional data that confirm the key role of nanoisland size in determining the number of bound DNA strands. In particular, larger islands of 100 × 100 nm led to considerably more binding of DNA (Fig. 3C). Indeed, the average fluorescence on large islands with 43500 ± 7800 counts (n = 200, N = 2) suggests that 10 DNA strands bound, which is higher than the calculated number of five closely packed flattened DNA hemispheres. One possible explanation is that the looped DNA might be compressed upon binding to the nanoisland which is in contrast to the less-compressible protein molecules where the amount scales directly with the island area. Furthermore, it cannot be ruled out that the larger islands size led to the partial binding of DNA strands at the fringes of the islands. Non-specific binding of DNA onto the surrounding PEG-coated non-target areas was low implying a high specific binding to the nanoislands (Supplementary Information, Fig. S-6).

**Figure 4:**
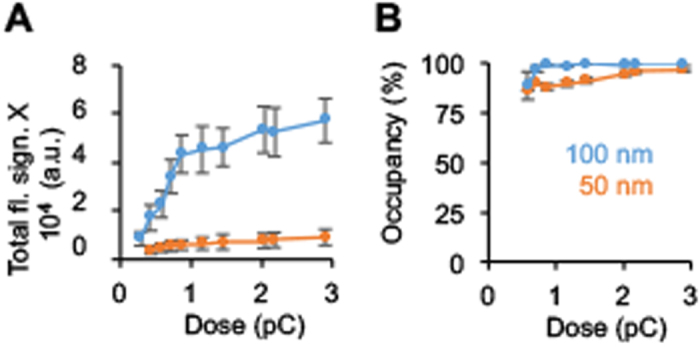
The apparent amount of Cy3-labeled DNA bound on carbon nanoislands depends on the carbon islands’ lateral size and their structural completeness which in turn is regulated by the e-dose. (**A**) The total fluorescence signal for 50 and 100 nm islands as a function of e-dose. The DNA was bound in the presence of 14 mM MgCl_2_. The averages and standard deviations were derived from two independent experiments comprising four data sets. The data are interpreted in the main body of the publication. (**B**) The occupancy of fluorescent DNA on avidin-coated 50 and 100 nm islands as a function of e-dose. Occupancy is defined as the percentage of nanoislands in a 10 × 10 array that are decorated with fluorophore-labeled DNA.

The amount of DNA strands on 50 nm islands did not go beyond the 1:1 stoichiometry when the island height was increased by higher e-doses. As shown in Fig. 4A, the brightness stayed almost constant over the dose range; a modest increase in fluorescence (Supplementary Information, Fig. S-7) is explained by the weakened quenching effect at larger distances. By contrast, 100 nm islands led to a much larger 5-fold increase in counts due to the formation of a contiguous and more adhesive carbon film up to a dose of 0.9 pC (Fig. 4A). The shallower increase above 0.9 pC is caused by more binding and, to a small extent, the height-induced de-repression of the ITO quenching effect. By comparison, the percentage of islands covered by DNA was less strongly influenced by e-dose. The coverage was 85% at the lowest doses and increased to reach almost 100% at the strongest e-beam (Fig. 4B).

The role of Mg^2+^ was explored as the divalent cation compacts DNA via charge screening and has a direct influence on the size of the DNA nanoball^38^. As expected, Mg^2+^ concentrations below the standard 14 mM which are increasing the diameter of the nanosphere did not strongly influence the average brightness on 50 nm island (Supplementary Information, Fig. S-8) as the number of DNA strands cannot be reduced below one. However, at 100 nm islands, fluorescence was strongly reduced (Supplementary Information, Fig. S-8) likely because the higher average size of the flattened DNA nanoballs caused fewer DNA strands to bind per islands. As additional explanation, less compaction could place more fluorophore-labeled DNA closer to the ITO leading to stronger quenching. The clear influence of Mg^2+^ concentration confirms the central concept that hydrodynamic DNA size defines the number of strands per island. The follow-on experiments were conducted at the standard concentration of 14 mM Mg^2+^.

### Further evidence for single molecule DNA array

**Figure 5:**
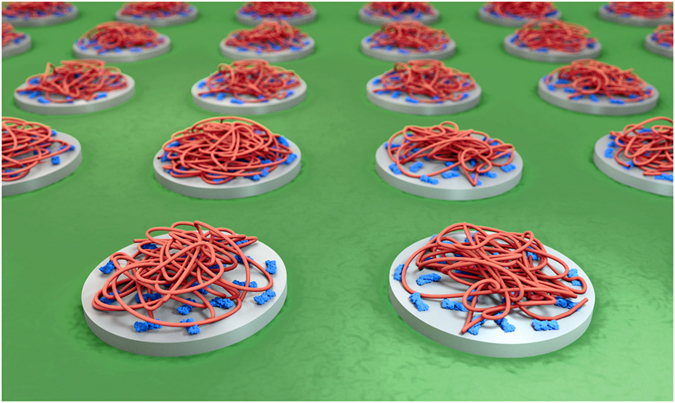
Schematic representation of an array of single DNA molecules (red) bound via positively charged avidin proteins (blue) onto carbon nanoislands (grey).

**Figure 6:**
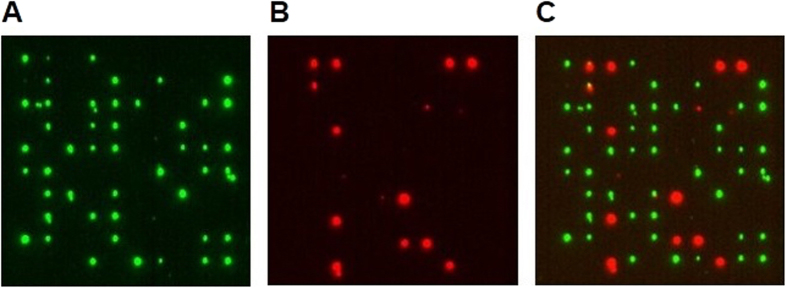
The lack of co-localization of Cy3 and Cy5-labeled DNA confirms that individual DNA strands bind onto avidin-coated 50 nm islands. Fluorescence microscopic images of an 10 × 10 island array written at 0.87 pC and scanned (**A**) in the Cy3 channel at 532 nm and (**B**) in the Cy5 channel at 646 nm. (**C**) Overlay of both fluorescence images.

As additional proof for the 1:1 stoichiometry of DNA strands per small nanoislands (Fig. 5), experiments on the anti-colocalization were conducted. In this assay, island arrays were incubated with a mixture of otherwise identical strands that are labeled with either Cy3 or Cy5 fluorophores. Provided that solely a single strand of DNA binds per islands, the islands should either have a Cy3 or Cy5 but not both fluorophore signals. The anti-colocalization of the two fluorescence signals was successfully established with fluorescence scanning in the Cy3 and Cy5 channel and in an overlay (Fig. 6). The results demonstrate that out of 58 DNA-covered islands, 45 were Cy3, 13 and Cy5 and only 1 had both fluorophores. A 1:1 stoichiometry of DNA molecule per islands was also established with DNA origami nanoplates of 50 × 50 nm onto small nanoislands (Supplementary Information, Figs S-10 to S-12). As expected, larger 100 nm islands with multiple DNA strands generated frequent co-localization of the Cy3 and Cy5 signal (Supplementary Information, Fig. S-9).

## Conclusions

This study has achieved the preparation of single-molecule arrays of DNA strands (Fig. 6). Evidence for the immobilization of single DNA strands per nanoisland is several-fold: (i) DNA strands on 50 nm islands had the same fluorescence signal strength as singulated DNA on plain substrate surfaces. (ii) When presented with Cy3 or Cy5-labeled DNA, 50 nm islands only bound a strand of one color. (iii) In line with the essential role of nanoisland size in limiting the number of DNA strands, multiple DNA molecules bound to larger 100 nm islands. (iv) In further support, 100 nm adsorbed fewer DNA strands when the diameter of the DNA nanoball was increased by lowering the Mg^2+^ concentration. At 50 nm islands, no gradual change was observed as the number of DNA strands cannot go below one.

The DNA arrays are set to enable a wide range of new experiments because DNA strands the size of genes or plasmids were immobilized on optically transparent substrates that can be read out with ultrasensitive fluorescence microscopy. In biophysics, the arrays are ideal to examine the behavior and structure of DNA at the single-molecule level in high lateral density. This includes studies on the interaction of the strands with DNA-binding proteins or anti-cancer DNA drugs. In cell biology, one exciting application is to overlay DNA arrays with cells to control uptake of individual genes to facilitate, for example, understanding the spread of antibiotic resistance. Of equal interest is to use DNA strands as anchoring sites for DNA aptamers that recognize cell-surface proteins and thereby alter their local position in the cellular membrane. In conclusion, the single-molecule DNA arrays of this study represent a general research platform.

## Methods

### Reagents

Indium tin oxide-coated glass slides (50 × 24 × 0.175 mm) with an ITO thickness of 17 ± 2 nm and a sheet resistance of 1200 ± 200 Ω/sq were obtained from Hans Tafelmaier Dünnschicht-Technik GmbH (Rosenheim, Germany). Me-O-PEG-(CH2)_3_-Si(OMe)_3_ with a MW of 460–590 D was bought from ABCR (#SIM6492.7, Karlsruhe, Germany). Avidin-Cy3 conjugate (#A4500-20) was purchased from USBiological (Swampscott, USA). Genomic DNA ULS Labeling Kit (#5190-0419) was supplied by Agilent Technologies (Vienna, Austria). Avidin (#A9275) and Amicon Ultra centrifugal filter untis (#Z648043-24EA) were obtained from Sigma-Aldrich Handels GmbH (Vienna, Austria). GelRed™ Nucleic Acid Gel Stain (#41003) was supplied by VWR International and Agarose NEEO ultra-quality (#2267.2) was purchased by Carl Roth GmbH (Karlsruhe, Germany). M13mp18 single-stranded DNA was purchased from NEB (Ipswich, USA). Conjugated DNA strand 5′-Biotin-TEG-/CTC GCT TCT GTC TAT CTT GGC-3′ were synthesized by Integrated DNA Technologies (Leuven, Belgium).

### PEG-Silanization

ITO surfaces were cleaned to remove organic contaminants. The slides were incubated in 10:90, 50:50, and 90:10 methanol: CHCl_3_ for 15 min each in an ultrasonic bath. After sonication, the slides were treated in basic Piranha (1:1:5 mixture of 30% ammonia and 30% hydrogen peroxide and water, freshly prepared, incubation for 40 min at 70 °C). After rinsing in deionized water and drying in a stream of nitrogen, the slides were plasma-oxidized in a Plasma System NANO (Diener electronic GmbH + Co. KG, Ebhausen, Germany) at 0.4 mbar for 2 min at 50 W. A layer of PEG silane was grafted onto the ITO surface by immersing the slides in 20 mM PEGsilane in anhydrous toluene containing 5% triethylamine as catalyst. The slides were incubated for 18 h at 60 °C, sonicated afterwards in toluene and ethanol for 5 min each to remove loosely bound PEG, rinsed with deionized water and dried in a stream of nitrogen. Silicon substrates were PEGylated following a similar procedure. Surfaces were first cleaned using ultra-sonication in acetone and then methanol for 15 min each, followed by incubation in basic Piranha (1:1:5 mixture of 30% ammonia and 30% hydrogen peroxide and water, freshly prepared) for 40 min at 70 °C, and plasma oxidation at 0.4 mbar for 5 min at 200 W. PEG was then grafted on the surface by overnight incubation in a 20 mM solution of PEG-silane in toluene supplemented with 0.08% conc. HCl as catalyst, followed by washing as described above.

### Electron Beam-Induced Deposition (EBID)

EBID features were written with an e-beam lithography system eLINE Plus (Raith GmbH, Dortmund, Germany). The eLINE Plus is equipped with a GEMINI field-emitter column that allows for an electron beam size of 1.6 nm for electron energies >3 keV. The background pressure during lithography is below 2 × 10^−5^ Torr in the sample chamber, and below 10^−9^ Torr at the cathode. The working distance during e-beam writing was typically chosen to be 10 mm. For a given beam current, typically around 3.5 pA, doses per EBID feature were adjusted via the integral dwell time of the beam in each target area. A series of arrays with systematically varied doses was written in one lithography run. The acceleration voltage was 20 kV and the aperture 10 μm. The e-beam intensity was an integer multiple of the minimal dose with a measured current 3.5 pA, an area dose 300 μC cm^−2^, and resulting dose of 0.029 pC for a 50 nm island. The e-beam lithography setup was operated in a Class 100 clean room.

### Atomic Force Microscopy

For analysis of nanoballs of DNA strands, a commercial atomic force microscope (Agilent Picoplus 5500, Agilent Technologies, Santa Clara, CA) equipped with a 90 μm closed loop scanner was used. The DNA sample m13mp18 (approximately 50 nM) dissolved in TAE buffer (40 mM Tris, 20 mM acetic acid, 1 mM EDTA, pH 8.2; supplemented with 14 mM MgCl_2_) was incubated onto freshly cleaved mica for 15 min and washed with buffer. AFM topographical images of DNA were acquired at RT using MSNL-10 cantilevers with a spring constant of 0.07 N m^−1^. Images were analyzed using Gwyddion software.

### Fluorescent Labeling of DNA

For generating Cy3 or Cy5-labeled DNA, the Genomic DNA ULS Labeling Kit was utilized. The kit contains a temperature-activated platinum-based compound which reacts with guanine bases in RNA and DNA. For the labeling reaction, a ratio of 1 μL ULS dye (Cy3 or Cy5) per 1 μg DNA was used. The appropriate amount of labeling master mix was added. The solution was heated up to 85 °C for at least 10 min before chilling it on ice for 3 min. Non-reacted Cy-dye was removed using KREApure purification columns. For additional purification, Amicon Ultra centrifugal filter units were used (100 kDa MWCO).

### Assays to Adsorb Protein or DNA onto Nanoisland Arrays

To examine the influence of writing parameters on biomolecule adsorption, arrays were incubated with a solution of avidin-Cy3 (2 μM, 0.1 × PBS buffer) for 30 min at RT. Subsequently, the slides were washed with 0.1 × PBS buffer and water, dried in a stream of nitrogen, and immediately analyzed using fluorescence microscopy. To generate avidin and DNA-decorated patterns, nanoislands were incubated with a solution of non-dye tagged avidin (2 μM, 0.1 × PBS) for 30 min at RT. In a second step, the avidin-coated islands were incubated with Cy3-labeled m13mp18 DNA or a mixture of Cy3 and Cy5-labeled DNA (1:1) in TAE buffer supplemented with MgCl_2_ (14 mM standard, or 10, 7, 4 mM) for 30 min.

### Fluorescence Microscopy

Fluorescence scanning of nanostructured and biofunctionalized ITO slides was conducted with an in-house developed fluorescence scanning device which is based on an inverted epifluorescence microscope (Axiovert 200, Zeiss, Oberkochen, Germany)^46^. For all measurements, a 100× objective (Zeiss, α Plan- FLUAR 100x/1.45) was used. Samples were mounted on a scanning stage (Märzhäuser, Wetzlar-Steindorf, Germany) and illuminated either with a diode-pumped solid-state laser with an emission line of 532 nm (Millennia IIs, Spectra Physics, Irvine, CA) or with a compact diode laser with an emission line of 642 nm (iBeam smart, Toptica photonics, Gräfeling, Germany). Images were taken with a Photometrics CoolSnap HQ digital camera (Roper Scientific, Trenton, NJ) (1392 × 1040-element CCD; pixel pitch, 6.45 mm × 6.45 mm; 12-bit; QE, 0.6) using a time-delayed integration mode. Image processing and analysis were performed with ImageJ (NIH, Beteshda, USA) and Excel (Microsoft, Redmond, USA). Samples were illuminated in epi-configuration with an average excitation intensity of ~0.48 kW/cm^2^ and an effective illumination time of 72 ms at 532 nm. Illumination time was set to 111 ms at 642 nm with an average excitation intensity of 0.57 kW/cm^2^.

To quantify the results of fluorescence scans, affinity *A*, specificity *S*, and occupancy were calculated. *A* relates to the amount of biomolecules bound per island. It was calculated from the difference of the average fluorescence of a 10 × 10 array of 100 × 100 nm^2^ or 50 nm circular islands covering a total area of 39 × 39 μm^2^, and the average background fluorescence from a proximal area without islands. Affinity thus represents the fluorescence of the island minus background. The carbon islands without adsorbed Cy-3-tagged biomolecule are non-fluorescent. By comparison, specificity *S* describes how many biomolecules bind per island in relation to the surrounding PEG surface of the same area. Specificity *S* is calculated from the ratio of the net fluorescence (affinity) over the background fluorescence, including a normalization factor to account for the different areas of nanoislands (100 × 10^4^ nm^2^) vs. surrounding PEG-coated area of 39 × 39 μm^2^. Finally, occupancy is defined as the percentage of nanoislands in a 10 × 10 array that are decorated with fluorophore-labeled biomolecules. An island was considered to be decorated with a fluorescence-labeled biomolecule when the fluorescence intensity of the island was higher than the fluorescence background plus three times the standard deviation of the background fluorescence.

## Additional Information

**How to cite this article:** Hager, R. *et al*. Arrays of Individual DNA Molecules on Nanopatterned Substrates. *Sci. Rep.* **7**, 42075; doi: 10.1038/srep42075 (2017).

**Publisher's note:** Springer Nature remains neutral with regard to jurisdictional claims in published maps and institutional affiliations.

## Supplementary Material

Supporting Information
