# Supplementary material for: Arrays of Individual DNA Molecules on Nanopatterned Substrates
Source: Sci Rep. 2017 Feb 13;7:42075. doi: 10.1038/srep42075 (PMC5304548; doi:10.1038/srep42075)
Supplement: Supporting Information [file srep42075-s1.pdf]

Supplementary Information

Arrays of Individual DNA Molecules on Nanopatterned Substrates

Roland Hager, Alma Halilovic, Jonathan R. Burns, Friedrich Schäffler, Stefan Howorka

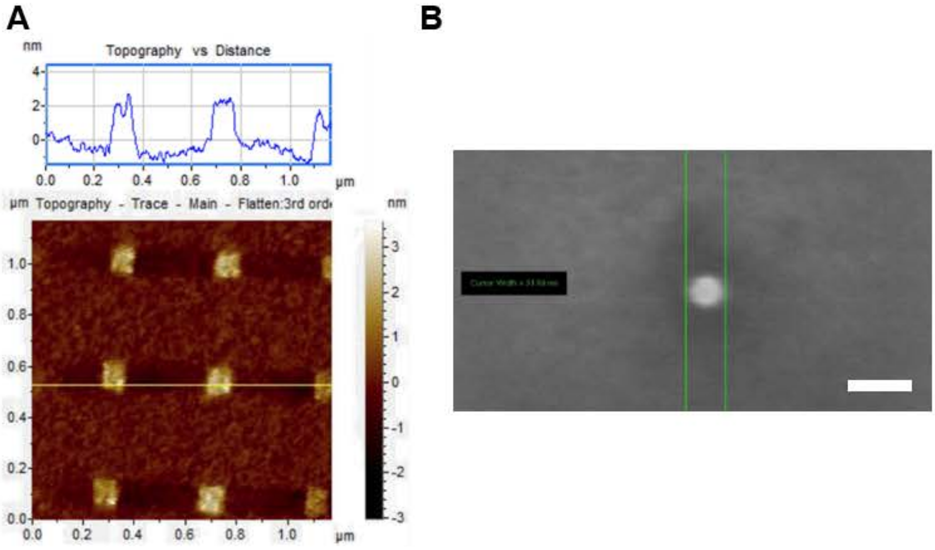

Figure S-1. Characterization of EBID structures. (A) AFM image and topographic information of 100 x 100 nm islands and (B) SEM image of circular nanoislands with a diameter of ~ 30 nm written on silicon substrates. The scale bar in B is 50 nm.

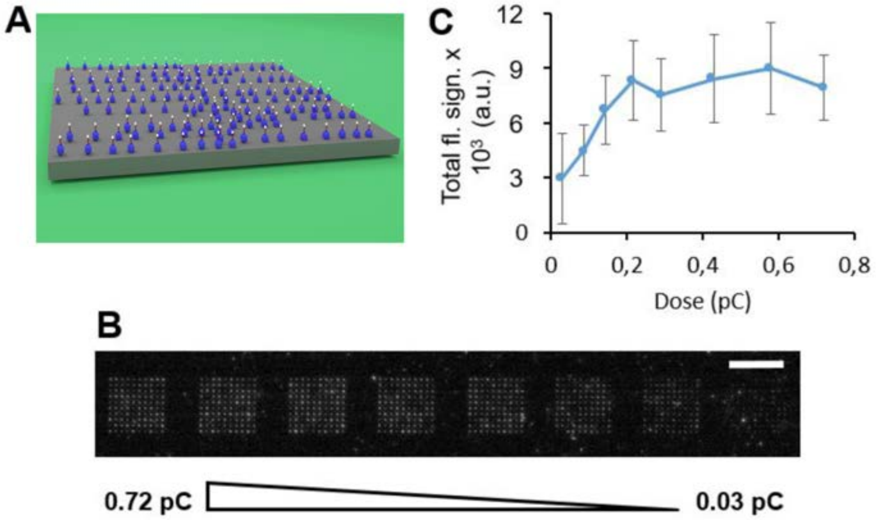

Figure S-2. The binding of avidin and biotin-DNA-Cy3 onto 300 x 300 nm carbon nanoisland arrays depends on the electronic writing dose. (A) Schematic illustration of carbon nanoislands decorated with unlabeled avidin and fluorescence-labeled DNA carrying a biotin tag. (B) Fluorescence microscopic image of a series of 10 x 10 arrays of 300 nm islands functionalized with avidin and biotin-DNA-Cy3. Islands within an array were written with the same electron dose while between the arrays the dose was varied from 0.03 to 0.72 pC. Scale bar is 32 µm. (C) Total fluorescence signal per island vs. electron dose, derived from the 300 nm island arrays in B.

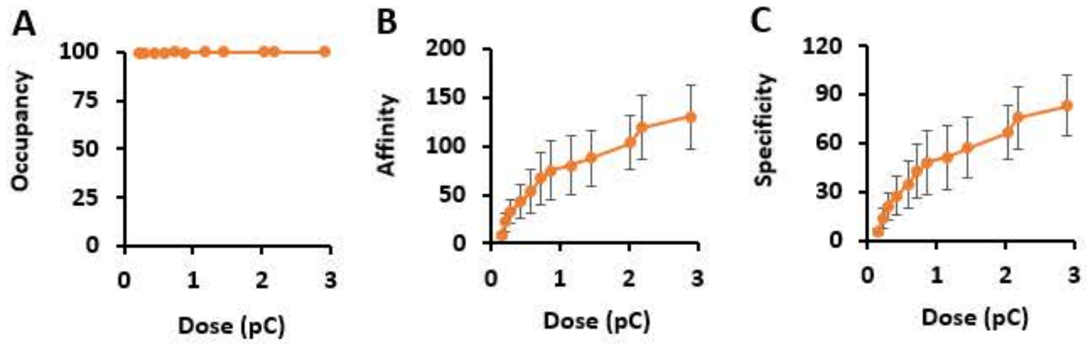

Figure S-3. The binding of avidin-cy3 onto 50 nm carbon nanoisland arrays depends on the electronic writing dose as shown for (A) mean occupancy of avidin-Cy3 islands, (B) affinity and (C) specificity of avidin-Cy3 binding. Occupancy, affinity, and specificity are defined in the methods section of the paper.

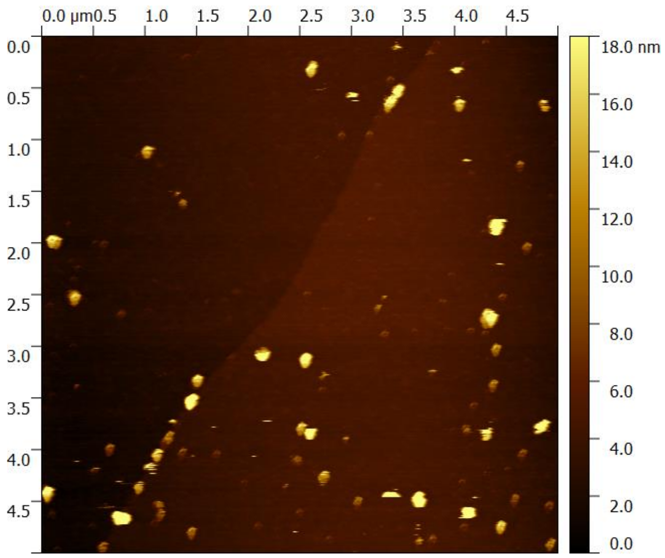

Figure S-4. AFM image of m13mp18 in TAE buffer supplemented with 14 mM $MgCl_2$. The measurement of 20 individual DNA nanoballs yielded a height of 14.9 ± 4.3 nm and a diameter of 119 ± 22 nm.

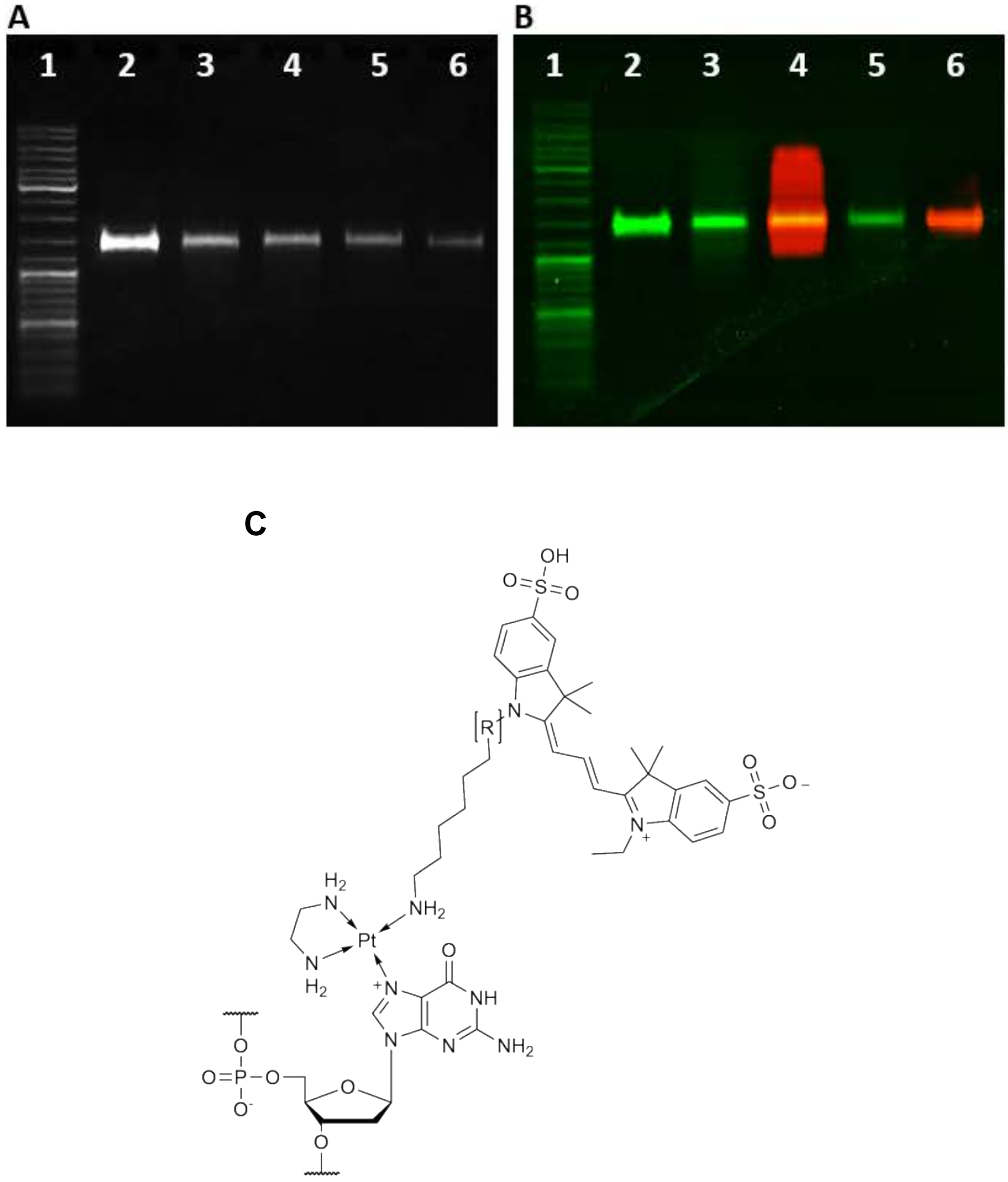

Figure S-5. Agarose gel electrophoresis of fluorescent-labeled DNA using the genomic DNA ULS labeling kit. Images were obtained by (A) UV transillumination and (B) overlaying fluorescence scans at 532 nm and 650 nm. Lanes: 1, GeneRuler DNA ladder mix SM0331 (reference bands: 3000, 1000 and 500 bp); 2, m13mp18 DNA; 3, m13mp18 DNA labeled with Cy3; 4, m13mp18 DNA labeled with Cy5; 5, m13mp18-Cy3 after purification via spin filters (100 kDa MWCO) to remove excess unbound fluorophores; 6, m13mp18-Cy5 after additional purification. (C) Chemical diagram of the guanine nucleotide modified at N7 with the Pt-containing ULS labeling regents with a Cy3 fluorophore. The chemical nature of residue R between the carbon linker and the fluorophore is unknown and has not been published by the commercial vendor.

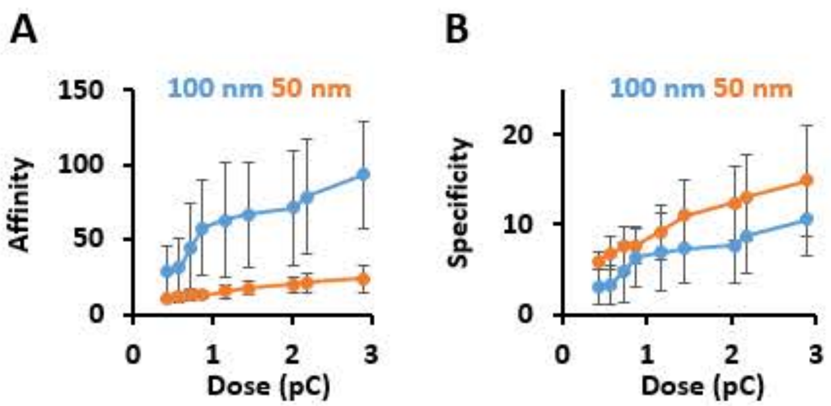

Figure S-6. Affinity (A) and specificity (B) for the binding of Cy3-labled m13mp18 DNA onto 50 and 100 nm avidin-coated nanoislands as a function of electron dose. Affinity and specificity are defined in the methods section of the paper.

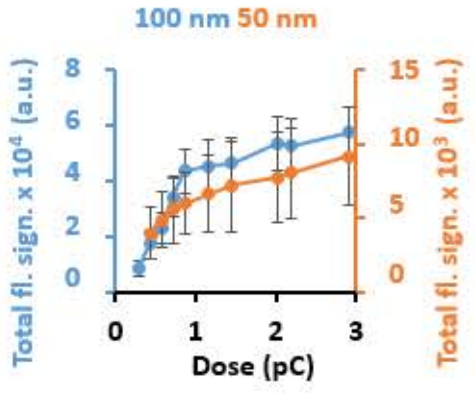

Figure S-7. Total fluorescence signal of Cy3-labled m13mp18 DNA bound to 50 and 100 nm avidin-coated nanoisland as a function electron dose.

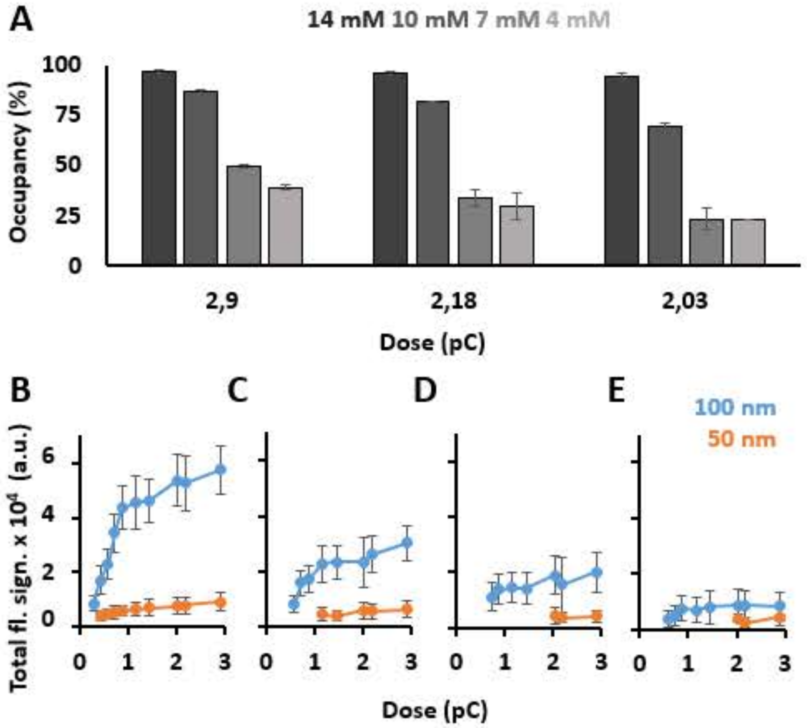

Figure S-8. The concentration of $MgCl_2$ influences the binding of Cy3-labled m13mp18 DNA onto avidin-coated nanoislands. (A) Mean occupancy for the binding of DNA onto nanoislands written at three selected electron doses as a function of (B-E) four $MgCl_2$ concentrations at 14 mM (B), 10 mM (C), 7 mM (D), and 4 mM (E). The plots also show the influence of the e-dose.

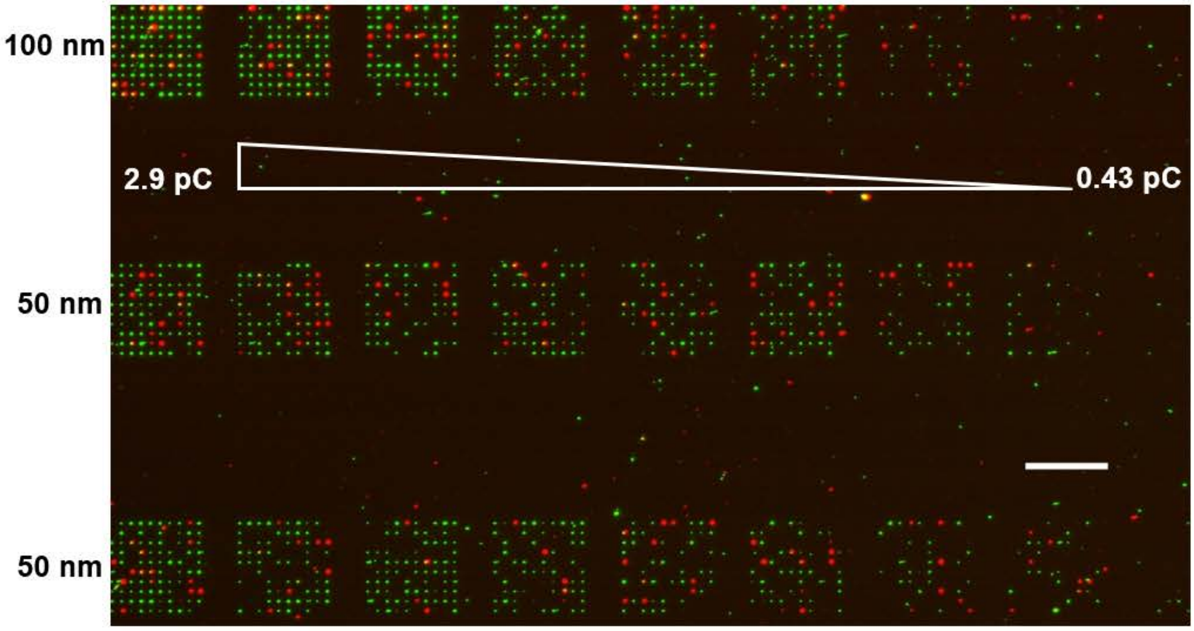

Figure S-9. Overlay of Cy3 and Cy5 channel fluorescence scans of avidin-coated 50 and 100 nm nanoisland arrays that were incubated with a mixture of Cy3-labeled and Cy5-labeled m13mp18 in TAE-buffer supplemented with 14 mM $MgCl_2$. The islands were either of square size with 100 x 100 nm or circular with a diameter of 50 nm. The islands within an 10 x 10 arrays were written with Islands within an array were written with the same electron dose while the dose was varied between the arrays was changes, as indicated. Scale bar, 30 μm.

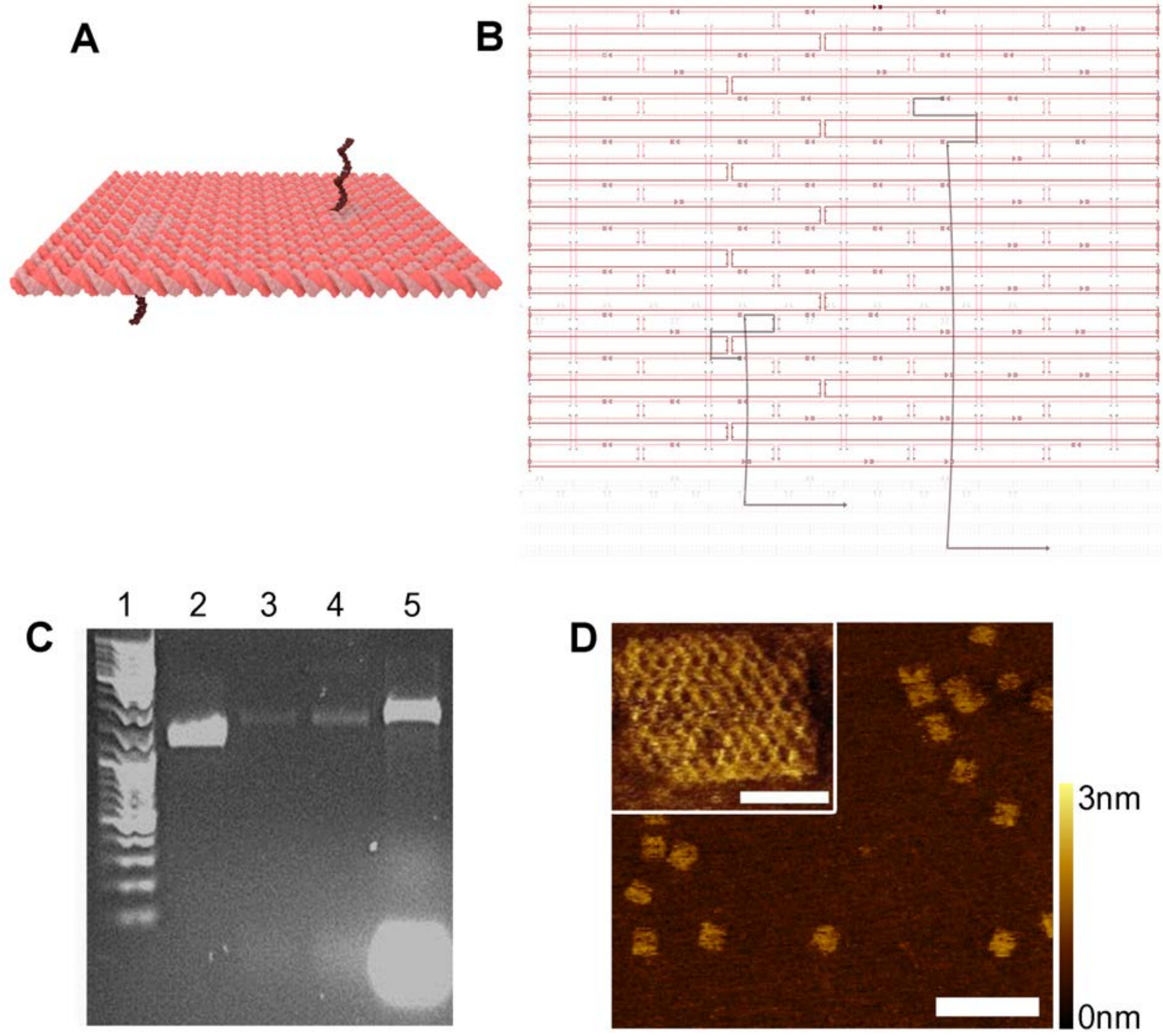

Figure S-10. Design, formation and characterization of DNA origami nanoplates. (A) Schematic drawing of a DNA nanoplate featuring a single duplex layer and lateral dimensions of 50 × 50 nm (blue and grey). The DNA plate features two unpaired DNA oligonucleotide (red) extensions on either side. (B) 2D map of the DNA origami nanoplate composed of parallel duplexes. The scaffold strand is shown in dark red, and staple strands are light red. 5' and 3' termini of the DNA strands are represented as squares and triangles, respectively. The sequences of the DNA oligonucleotides are shown in Table S-1. The procedure to assemble and purify the DNA nanoplates is described in Hager et al *Small*, 2016, 12, 2877-2884. (C) Gel electropherogram of DNA nanoplates: lane 1, 1 kbp DNA ladder; lane 2, M13mp18 scaffold strand; lanes 3 and 4, purified DNA origami nanoplates; lane 5, DNA origami plate before purification with excess staple strands. (D) AFM image of DNA nanoplates adsorbed on mica, scale bar, 200 nm. Inset, higher resolution image of plate, scale bar 25 nm.

Table S-1. Sequences of DNA Staple Strands

Strand Name	Sequence, 5' → 3'
0[102]	AAAAACCCGGCAAAATCCCTTATCGGTCCAC
15[104]	TAAAAATTTAGCAAAATTAAGCAATAGCTATAT
7[5]	GCTTTGAGGACAACGAAAGAGG
2[55]	AACAACTATTGCTAAACAACTTTCCAACGCCTGTAGC ATTCCACAGAC
6[87]	GAGGATCCAGCTCGAATTCGTAATAATTGCGTTGCG CTCACAACGCGCG
6[154]	TTTTCCCAGTCCACACAACATA
20[134]	ATCGCGTGCGGATTGCATCAAAAAGATTAAGAGG
12[55]	CAAGAGGATGAAAATATTTAAATTCATCGCCT
8[103]	ACCGCTTCGATAGGTCACGTTGGTAGCGAGTA
4[70]	TTCCAGTGAGTTAAAGGCTACCGCCGGGCGC
2[103]	GCTGGTTTGCGGTTTGCGTATTGGTGAGCTAA
9[41]	AGCGATTGTGTCGAAATCCGCGACCGAACTGACCAA CTTCAGATGAAC
10[23]	ATGTTACTACTAAAACACTCATCTGAAGGCAC
9[121]	CGCATCGTCGCACTCCAGCCAGCAGGGGGATG
2[154]	CTGATTGCCCTTGTTGTTCCAG
1[88]	TCCGAAATGTCTATCAGAGTTTCGTCACCAGT
13[5]	TCATCAAGAGTGGTTTAATTTC
18[23]	TATCATAAAACATTATTACAGGTAGAAGAAAA
11[120]	TTTTAACCATCAACATTAAATGTGGTAGATGGG
17[137]	TCAATTCCATTCCATATAACAGTTTTTAAATATGCAAC TACTTCAAAT+C93
6[55]	CCCTCAGCCGCTGAGGCTTGCAGGCGGGAAAC
18[55]	AAAGGAATGAGATTTAGGAATACCTACCAAAA
15[40]	AAGAACTGCCAGTCAGGACGTTGGGAAAGATTCATC AGTTTACGAGGC
16[71]	ATCGGTTGACATTCAACTAGCAAAATTTCATGCAGAT ACAAAGAGGTC
6[71]	TTTTGCGGGGAAGGGCGATCGGTGGCAAAGCG
6[23]	GGTAGCAAACCATCGCCCACGCATACAGCTTG
14[71]	AATAAGGCTAATACTTTTGCGGGAAAAGCTAA
11[137]	GCCATCAATGAACGGTAATCGTAGGTCATTGC
21[57]	AGTTCAGACCTTTAATTGTTTGCGAAGTCTCCTTTTG ATTAACGCCA
11[5]	AGGCGCAGACGCTGGCTGACCT
6[39]	ACAGCATCAGGAAGTTTCCATTAATACGTAATGCCA CTACTTGACCCCC
1[136]	GGGTTGAGTCACCGCCTGGCCCTGTCTTTTCAC
13[120]	GGCTATCAAAACTAGCATGTCAATAGCTCATT
8[23]	CAACCTAATAAAGACTTTTTCATGGGAACGAG
16[55]	ACATTATAGCTCATTATGACCCTGTTGCCCTG
4[103]	CTCACATTCATGGTCATAGCTGTTTTGCATGC
16[154]	GTAGCATTAACCTGAGTAATGT
8[55]	CTGTAAAAACGGGCGCAACTGTTGGATCGTCA
6[103]	CTGCAGGTTACGCCAGCTGGCGAATTTCCGGC
2[23]	TCACGTTGGACGTTAGTAAATGAATAGTTAGCGTAA CGATCTAAAGTTTTG

14[154]	GTAGGTAAAGATCTGGAGCAAA
16[87]	CAGAGCATGAAGCCTTTATTTCAAATATTCAAC
0[39]	AGCCCTCATTTTCTGTATGGGATTAAGGAATTGCGA ATAATTGTATCGG
3[137]	CAGTGAGCATAAAGTGTAAAGCCTATCCGCTCACAA TTCACGACGTT
4[23]	ATACCGATCAAAAGGAGCCTTTAATAATTTTT+C23
19[121]	CAACATGTGATTCCCAATTCTGCTGGGGCGC
3[41]	TTTATCAGAGGTGAATTTCTTAAAACCGATATATTCG GTAGCGAAAG
0[154]	TTTGGAACAAGAGTCCACTATTAAAGACCGAGATA
13[137]	CTGAGAGTTCAAAAGGGTGAGAAATTTTAAA
21[86]	AAAAATCAGGAAGCAAACTCCAACAGGGATGGCT
21[5]	TACTGCGGAATCGTCATAAATATTCATAAAATGTT
15[121]	CTCATATAGGCCGGAGACAGTCATCTACAAA
0[71]	ACAAACTAAACAGTTTCAGGTTTGATCCTCGG
18[154]	TCTGGAAGTTTTACTAATAGTA
1[5]	TCGTCTTTCCAAAAATCTCCAA
4[119]	CTAATGAGGCGCCAGGGTGGTTTTAGAGAGTTG
10[154]	CGTCTGGCCTTAGTTTGAGGGG
14[23]	CTTGAGATAATCTTGACAAGAACCAGACCAGG
21[105]	TGACTATTATAGTCAGAAGCAAATTTAATTCGAGCTT CAGCTGTAGCT
7[121]	TGCTGCAACGGCCAGTGCCAAGCTCCTGTGTGAAAT TGTTGGGGTGC
10[55]	GATAAATTATACCAAGCGCGAAACCATTCAGG
8[87]	GAAACCAGCGGGCCTCTTCGCTATCGACTCTA
19[104]	AATATAATAAGCGAACCAGACCGGTCTTTACCC
18[39]	ATAGTAAGAGAGGCTTTTGCAAAACAGAGGGG
14[55]	ACGAGAAAAATCAACGTAACAAAGTGTATAAG
10[103]	ACAACCCGAAATTTTTGTTAAATCCATATGTACCCCG GTTAGCTATTT
19[72]	ATTTTTGCGTCAGGATTAGAGAGTAAAACGAGAATG ACCATAAATC
10[71]	ATTTGTATGTAAACGTTAATATTTCCAAAAAC
15[5]	AACTTTAATCATAATAAAACGA
19[88]	TAGAGCTTTTTGACCATTAGATACTGGTCAATAACCT GTTTAAAGCCT
5[5]	CAATGACAACACGGCTACAGAG
12[87]	GAAAAGCCTGTTAAAATTCGCATTTCGGATTC
4[154]	CGAGCCGGAAGACGGGCAACAG
9[137]	ATCTGCCCCTGTAGCCAGCTTTCAATAGGAAC
17[120]	GAGCTGAAAGGCAAGGCAAAGAATTTTAGAACC
8[71]	CCATTCGCAAAGTACAACGACGGCAACAAGAG
13[104]	TTGAGAGAAATCACCATCAATATGCGCAAGGA
9[5]	CAAAAGAATACTAGCCGGAACG
10[87]	TCCGTGGGGGATTGACCGTAATGGTGGTGCCG
19[5]	CAGACGACGATATAGCGTCCAA
20[23]	TAGACTGGAAAAACCAAAATAGCGAGCAACAC
8[154]	ACGACGACAGTTAACGCCAGGG
17[105]	TTTCATTGAACGAGTAGATTTAGAATTGCTG
12[154]	CAAGAGAATCGAAAATAATTCG

2[71]	AGTGAGAAGCATTAATGAATCGGCCTGCCCGCT
14[86]	CGTTCTATAATGCCGGAGAGGGTGATAATCA
4[55]	CTGCTTTCGCTTGTCGTGCCAGCTTAGAAAGG
12[23]	CGCATAGGGTCAATCATAAGGGAACCTGCTCC
20[154]	AAGCCCGAAAGAAAGTACGGTG
3[89]	GGGAGAGGCCCCAGCAGGCGAAAATGGTGGT
20[39]	GTAATAGTTGAATCCCCCTCAAATGCTTTAAAC
12[71]	AGGAAGATCTGCTCATTCATAAATGCTGAGTG
3[5]	AAAAAAGGCTCAGTTGCGCCGA
17[5]	ACTAACGGAACCCCTCGTTTAC
2[118]	CAGCAAGAAATCAAAAGAATAGCACGTGGACTCCAA<br>CGTCAAAGGGCG
6[135]	GTAAAACGAGGCGATTAAGTTGGGATCGGCCTCAG<br>GAAGATAACCGTGC
15[136]	TGCAATGCATCCAATAAATCATACAAGGTGGCA
12[38]	GGTGTACGGATATTCATTACCCACACCAGAACGAGT<br>AGTGCGATTTT
16[23]	ATCTACGTTTGTGAATTACCTTATAAATTGGG

**Functional DNA strands:**		**Replaces strand:**
16[55]Biotin	ACATTATAGCTCATTATGACCCTGTTGCCCTGTTTTG<br>CCAAGATAGACAGAAGCGAG-TEG-Biotin	16[55]
4[103]Biotin	CTCACATTCATGGTCATAGCTGTTTTGCATGCTTTTG<br>CCAAGATAGACAGAAGCGAG-TEG-Biotin	4[103]

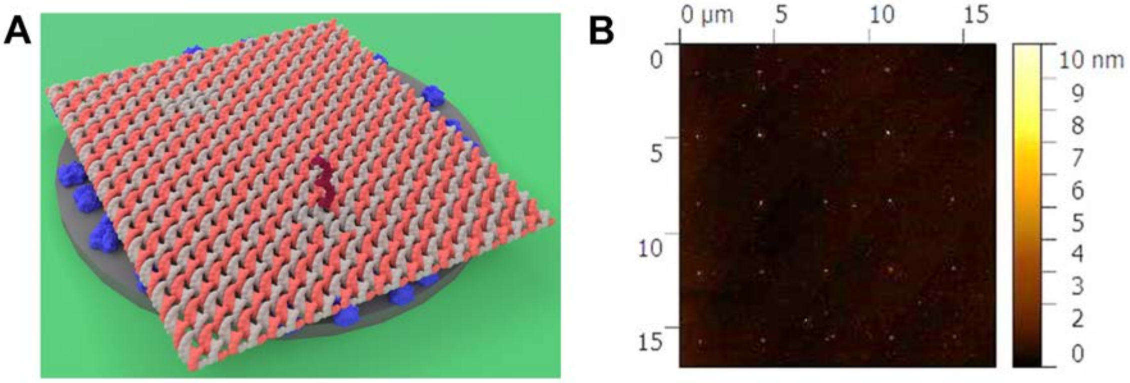

Figure S-11. (A) Schematic drawing of a carbon nanoisland decorated with avidin and DNA origami nanoplates. (B) AFM analysis of 50 nm EBID nanoislands written at a dose of 1.16 pC on silicon substrates. The islands were decorated with avidin and electrostatically bound DNA origami nanoplates. The island height is 8.2 ± 3.6 relative to the surrounding silicon surface. The mean height of the EBID structures without avidin or DNA origami is 3.3 ± 0.5 nm.

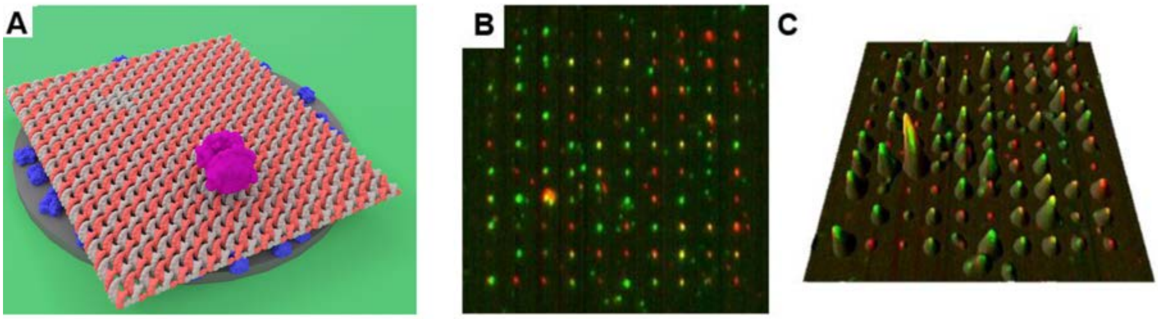

Figure S-12. (A) Schematic drawing of a carbon nanoisland decorated with avidin and a DNA origami nanoplate that carries at its top a fluorophore-labeled streptavidin that is tethered via DNA duplexes to the DNA nanoplate. (B,C) Fluorescence microscopic images of a 10 x 10 island array of nanoislands written at 2.03 pC that were incubated with a mixture of 50 x 50 nm DNA origami nanoplates carrying either Cy3 or Atto 655-labeled streptavidin in order to achieve a stoichiometry of 1 nanoplate per nanoisland. The nanoislands with adsorbed avidin were incubated for 30 min at room temperature with 7 nM DNA nanoplates in TAE buffer, pH 8.0 supplemented with 14 mM $MgCl_2$, followed by washing with the TAE-$MgCl_2$ buffer. To load the fluorescently labeled streptavidin-DNA conjugate onto the DNA plates, a 2 µM solution of the conjugate in PBS buffer was added to the array and incubated for 45 min followed by washing with PBS buffer and deionized water. The conjugate was composed of streptavidin and biotinylated oligo B (5'-BiotinTEG/CTC GCT TCT GTC TAT CTT GGC-3') at a molar ratio of 1:4 so that the protein-DNA conjugate can bind to the three complementary oligo A strands of the DNA nanoplate. For fluorescence microscopic analysis of the array, scanning was performed in Cy3 channel at 532 nm in the Cy5 channel at 646 nm. Shown is the overlay of both fluorescence images. Panel C shows the information of panel B but with the fluorescence intensity indicated by the peak height.
